# Molecular and Serological Screening of West Nile and Usutu Virus in Birds and Humans: A Sentinel Study in Finland

**DOI:** 10.1002/vms3.71125

**Published:** 2026-08-07

**Authors:** Ruut Joensuu, Maija T. Suvanto, Sanna Sainmaa, Viktor Olander, Roope Nykänen, Essi M. Korhonen, Anne J. Jääskeläinen

**Affiliations:** ^1^ Department of Veterinary Biosciences Faculty of Veterinary Medicine University of Helsinki Helsinki Finland; ^2^ Department of Virology Faculty of Medicine University of Helsinki Helsinki Finland; ^3^ Department of Geosciences and Geography Faculty of Science University of Helsinki Helsinki Finland; ^4^ Korkeasaari Zoo Helsinki Finland; ^5^ HUS Diagnostic Center Clinical Microbiology University of Helsinki and Helsinki University Hospital Helsinki Finland

**Keywords:** diagnostic tools, RT‐qPCR, Usutu virus, USUV, West Nile virus, WNV

## Abstract

**Background:**

The northward expansion of mosquito‐borne viruses such as West Nile virus (WNV) and Usutu virus (USUV) in Europe, driven partly by climate‐related changes affecting vectors and hosts, poses an increasing threat to human and animal health. Reliable diagnostic tools are therefore essential for early detection and effective surveillance.

**Objectives:**

This study aimed to develop and evaluate sensitive molecular assays for WNV and USUV, apply them to screen wild birds and human patients in Finland, assess bird exposure through serology, and evaluate national preparedness for emerging arboviral threats.

**Methods:**

Real‐time PCR assays for WNV and USUV nucleic acids were developed, optimized and evaluated for the detection of WNV and USUV nucleic acids using bird and human samples. Bird samples were also tested for anti‐WNV antibodies using an in‐house immunofluorescence assay (IFA).

**Results and Conclusions:**

The real‐time PCR assays showed high sensitivity, with limits of detection of 13.0 copies per reaction for WNV and 14.6 copies per reaction for USUV. A total of 163 deceased birds and 337 human serum samples collected during the summer months from patients with suspected acute infections were screened. No WNV or USUV nucleic acids were detected, and bird samples tested negative for WNV antibodies. However, given the active migratory bird flyways from Africa and Southern/Central Europe, and recent detections in neighbouring countries, the introduction of WNV into Finland appears likely. Continued surveillance and the availability of efficient diagnostic tools remain critical for preparedness and early response.

## Graphical abstract

We developed sensitive real‐time PCR assays for West Nile virus and Usutu virus to screen bird and human samples in Finland. Although no positive samples were detected, active migratory flyways and recent cases in neighbouring countries highlight the risk of future introduction and the need for continued surveillance.

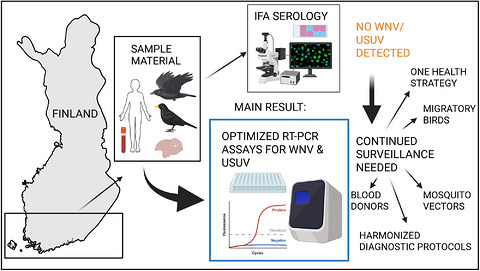

**Abbreviations**
BSA: bovine serum albumin
IFA: immunofluorescence assay
LOD: limit of detection
PCR: polymerase chain reaction
RT‐PCR: real‐time polymerase chain reaction
SINV: Sindbis virus
USUV: Usutu virus
WNF: West Nile fever
WNND: West Nile neuroinvasive disease
WNV: West Nile virus

## 1 Introduction

West Nile (WNV) and Usutu virus (USUV) are arthropod‐borne, single‐stranded RNA viruses of the *Flaviviridae* family, genus *Orthoflavivirus*, and members of the Japanese encephalitis virus serocomplex (Kuno et al. 1998; Weissenböck et al. 2002; Habarugira et al. 2020). Both viruses can cause disease in humans and animals. WNV, originally from sub‐Saharan Africa, is classified into nine distinct lineages, with lineage 1 and 2 being most relevant to human and animal health. Lineage 1 is globally distributed and has been circulating in Europe since the first report in France in the 1960s, while lineage 2 became established in Europe in the 2000s and is now responsible for most human infections across Central and Southern Europe (Sule et al. 2018). USUV was first detected in Italy in 1996 and includes eight distinct lineages (Africa 1–3, Europe 1–5) with Europe 2 being the most prevalent in Southern and Central Europe (Vilibic‐Cavlek et al. 2019; Weissenböck et al. 2013; Zecchin et al. 2021). Both viruses are progressively expanding their geographical range in Europe (Vilibic‐Cavlek et al. 2019; Zeller and Schuffenecker 2004; Sikkema et al. 2020).

In humans, WNV infection is often asymptomatic (80%), but around 20% develop West Nile fever (WNF), and approximately 1% progress to West Nile neuroinvasive disease (WNND) (Zannoli and Sambri 2019; Jani et al. 2022; Schwarz and Long 2023). Symptoms of WNF include headache, fever, nausea, muscle and joint pain, while WNND can lead to encephalitis, acute flaccid paralysis and meningitis, with a fatality rate of 10% (Jani et al. 2022; Gamino and Höfle 2013). In horses, WNV can cause neurological signs and behavioural changes (Schwarz and Long 2023; Vilibic‐Cavlek et al. 2020). Most USUV infections in humans are asymptomatic, though rare cases with febrile illness or neurological symptoms have been reported, primarily in immunocompromised or elderly individuals (Pecorari et al. 2009; Angeloni et al. 2023). USUV can also cause high mortality in wild birds (Weissenböck et al. 2002). In addition to mosquito bites, WNV and USUV can be transmitted through faecal‐oral and contact‐based routes, as well as via blood transfusions and organ transplantation (Habarugira et al. 2020; Schwarz and Long 2023; Garzon Jimenez et al. 2021).

Both viruses are maintained in an enzootic cycle between ornithophilic mosquitoes—primarily *Culex pipiens* complex and *Culex torrentium* in Europe—and viremic wild birds, which serve as amplifying hosts and long‐range vectors (Habarugira et al. 2020; Gaibani and Rossini 2017; Roesch et al. 2019; Vidaña et al. 2020; Krambrich et al. 2024). Avian susceptibility to WNV varies; corvids such as crows and jays are highly susceptible, while house sparrows show greater tolerance (Zannoli and Sambri 2019; Gamino and Höfle 2013; Nemeth et al. 2009). The Eurasian blackbird (*Turdus merula*) and great grey owl (*Strix nebulosa*) are notably susceptible to USUV (Nikolay 2015; Störk et al. 2021). Humans, horses and other vertebrates are considered dead‐end or incidental hosts due to low viremia levels (Schwarz and Long 2023; Laidoudi et al. 2023; Agliani et al. 2023).

In Europe, WNV cases in humans and/ or horses have been reported in 19 countries, including Italy, France, Germany, the Netherlands, and Spain; however, USUV infection is not a notifiable disease in Europe (European Centre for Disease Prevention and Control (ECDC) 2023). In Finland, WNV diagnostics rely on both molecular assays and serology. However, in regions where other flaviviruses—such as tick‐borne encephalitis virus (TBEV)—are endemic and vaccination is common, cross‐reactivity in serological testing complicates accurate diagnosis (Garzon Jimenez et al. 2021). As WNV continues to expand northward, Finland remains at risk of reporting its first case. Increased risk poses challenges for public and animal health, particularly in human blood donation and transplantation safety. Surveillance of migratory birds is essential, given the active flyways connecting Finland to Africa and Southern Europe (Olowu et al. 2023). In this study, we aimed to develop and validate diagnostic methods for WNV and USUV nucleic acid detection and to screen bird and human samples from Finland for early evidence of virus circulation.

## 2 Materials and Methods

### 2.1 Aim of the Study

The aim of this study was to develop specific diagnostic methods for the detection of WNV and USUV nucleic acids and to investigate the evidence of virus circulation and seroprevalence of these viruses in wild birds in Finland.

### 2.2 WNV‐ and USUV‐RT‐PCR

Primers and probes for WNV and USUV real‐time RT‐PCRs (WNV: 5′‐untranslated region (5′UTR) and capsid (C) gene, 29; USUV: non‐structural protein 5 (NS5) gene, 30) were taken from previously published methods (Linke et al. 2007; Cavrini et al. 2011) and optimized using Superscript III Platinum One‐step qRT‐PCR System (Invitrogen, Carlsbad, CA, USA) for diagnostic settings. The WNV‐RT‐qPCR was carried out using 320 nm of primers (modified from (Linke et al. 2007); WNV‐MOD‐FW, 5′‐YCYGYGTGARCTGACARACTTAGT‐3′, WNV‐MOD‐RV 5′‐GCGTTTYWGCATATTGACRGCC‐3′), 60 nm of both probes [FAM‐ATCYCGATGTCYAAGAAAC‐MGBNFQ and FAM‐TCRATGTCYAAGAAACCA‐MGBNFQ; this study], and USUV‐RT‐qPCR using 850 nm of primers of USUV‐F (5′‐AAAAATGTACGCGGATGACACA‐3′; 30) and USUV‐R (5′‐TTTGGCCTCGTTGTCAAGATC‐3′; 30) and 250 nm of USUV‐probe (VIC‐CGGCTGGGACACCCGGATAACC‐MGBNFQ; modified from 30) with Superscript III Platinum One‐step qRT‐PCR System (Invitrogen). Cycling conditions were adjusted for Invitrogen‐kit and those were 30 min at 50°C followed by 2 min at 95°C, and 45 cycles of 15 s at 95°C followed by 50 s at 60°C in a MX3005P QPCR System (Agilent Technologies), and a final template amount was 7 µL. Sensitivity limit of detection (LOD), and intra‐assay repeatability/reproducibility were determined using commercial quantified plasmid controls of WNV (strain B956) and USUV (isolate Budapest). For LOD determination, Probit Analysis (SPSS Statistics, IBM, Helsinki, Finland; confidence interval (CI) 95%) was used, and for LOD and intra‐assay studies, at least 10 parallel reactions were carried out. Specificity study was carried out for WNV‐RT‐qPCR, as the USUV‐RT‐qPCR was previously validated by Cavrini et al. (2011, 30).

### 2.3 Controls and Viral Nucleic Acids

For cross‐testing of WNV‐RT‐qPCR, several extracted nucleic acids were tested: dengue viruses 1–4 (DENV1–4; Zeptometrix Corporation, Buffalo, New York, USA, and Robert Koch Institute (RKI), Germany), Zika virus (strain MR766; ZIKV), yellow fever virus (strains 17D and Asibi, RKI; YFV), TBEV (subtypes European, Siberian, and Far Eastern, in‐house), and chikungunya viruses (strains Caribbean, European Virus Archive (EVAg) and Ross, in‐house; CHIKV). Ten external quality control samples (EQA samples) from EVD‐Labnet comprising nucleic acids of Toscana virus (lineage A and B), TBEV, USUV, WNV lineage 1 (strain UVE/WNV/2001/FR/DON2001), WNV lineage 2 (strain B956), and negative plasma samples (Reusken et al. 2019) were tested using WNV‐RT‐qPCR (Table 1).

**TABLE 1:** Bird species and their respective sample numbers included in the analysis.

Bird species	Scientific name	Number of bird samples
Barnacle Goose	*Branta leucopsis*	4
Goosander	*Mergus merganser*	1
Common Gull	*Larus canus*	2
Hooded Crow	*Corvus corone cornix*	16
Wood Pigeon	*Columba palumbus*	19
Eurasian Sparrowhawk	*Accipiter nisus*	5
Rock Dove	*Columba livia domestica*	3
European Magpie	*Pica pica*	3
Fieldfare	*Turdus pilaris*	20
Great Spotted Woodpecker	*Dendrocopos major*	2
Eurasian Blackbird	*Turdus merula*	16
Common Kestrel	*Falco tinnunculus*	1
Bohemian Waxwing	*Bombycilla garrulus*	1
Tawny Owl	*Strix aluco*	2
Tengmalm's owl	*Aegolius funereus*	1
Eurasian Jackdaw	*Corvus monedula*	2
European Nightjar	*Caprimulgus europaeus*	1
Short‐eared Owl	*Asio flammeus*	1
Eurasian Hobby	*Falco subbuteo*	1
Common Swift	*Apus apus*	17
Eurasian Bullfinch	*Pyrrhula pyrrhula*	3
Great Tit	*Parus major*	5
Eurasian Siskin	*Spinus spinus*	5
Common Pheasant	*Phasianus colchicus*	2
Chaffinch	*Fringilla coelebs*	7
White Wagtail	*Motacilla alba*	2
Blue Tit	*Cyanistes caeruleus*	3
House Sparrow	*Passer domesticus*	10
Coal Tit	*Periparus ater*	3
Willow Warbler	*Phylloscopus trochilus*	1
Northern Goshawk	*Astur gentilis*	1
Goldcrest	*Regulus regulus*	1
Common Goldeneye	*Bucephala clangula*	1
European Goldfinch	*Carduelis carduelis*	1

### 2.4 Indirect Immunofluorescence Assay (IFA) for Bird Samples

First, we prepared IFA slides for WNV antibody detection. Vero E6 cells were cultured in T‐75 flasks and infected with WNV (T) Egypt P‐3689 at approximately 80%–90% confluency. After an hour of incubation at 37°C, 20 mL of MEM (Sigma‐Aldrich, United States) containing 2% FBS and antibiotics (penicillin‐streptomycin) was added. The infected cells were incubated for 3 days to allow for viral replication. On the third day, both infected and uninfected control cells were detached using Trypsin‐EDTA, washed in PBS, and resuspended in PBS at a concentration of approximately 6 mL per confluent flask. The cell suspension was thoroughly mixed and spotted onto glass IFA slides (25 µL per well). The slides were air‐dried overnight in a laminar flow hood. For fixation, the dried slides were placed in slide racks, submerged in pre‐chilled acetone at −20°C for 7 min, and subsequently stored at −80°C until use. The quality of prepared IFA slides was validated using a known positive control, and titration results were recorded for assay consistency.

Screening for anti‐WNV IgG antibodies was performed using an in‐house IFA assay adapted from (Vene et al. 1995). Known WNV IgG‐positive samples served as positive controls in the IFA tests. The bird blood samples were diluted from 1 cm^2^ of filter paper in 1 mL Dulbecco + BSA and kept at +4°C overnight. The next day, 20 µL of the sample was added to the IFA slide well and incubated in a humid chamber at 37°C for 60 min. The slides were then washed for 3 × 5 min using PBS. Subsequently, anti‐chicken FITC IgG conjugate, diluted to 1:50, was added to the slides and incubated at 37°C in a moist chamber for 30 min. Following incubation, the slides were washed for 3 × 5 min in PBS and 1 × 5 min in Milli‐Q water. After drying, the slides were mounted with Immu‐Mount mounting media (Thermo Scientific, United States) and observed under a UV microscope.

### 2.5 Bird Samples

We obtained bird samples (*N* = 163) from Korkeasaari Zoo Wildlife Hospital, including naturally deceased, diseased and euthanized birds representing both resident and migratory species (*N* = 34) collected in 2020 (Table 1). Blood samples were collected post‐mortem onto filter paper and stored at −20°C until laboratory analyses. In addition, carcasses were stored at −80°C until processing. During brain dissection, brain tissue samples were aseptically collected and stored at −80°C until RNA extraction. Following extraction, RNA samples were stored at −80°C until further analyses.

### 2.6 Human Serum Samples

Human serum samples (*N* = 337; June–October 2021, Finland; HUS Diagnostic Center, Helsinki, Finland), were retrospectively collected from archived samples from patients suspected of an acute infection during summer and autumn months. These samples were tested for WNV and USUV nucleic acids using WNV‐ and USUV‐RT‐qPCR. The RNA was extracted using the MagNa Pure LC system and kits (Total Nucleic Acid Kit, Roche, Espoo, Finland), and EasyMag (bioMérieux, Marcy l'Étoile, France), respectively, following the manufacturers’ instructions using 200 µL of original sample and an elution volume of 50 µL.

## 3 Results

### 3.1 WNV‐RT‐qPCR and USUV‐RT‐qPCR

The specificity of the WNV‐RT‐qPCR assay was excellent (Table 2). Different WNV strains with different virus quantities were all detected (Table 2). The EVD‐Labnet EQA samples were all correctly detected (10/10, 100%). The LOD was 13 copies per reaction for WNV‐B956 using Probit Analysis (SPSS Statistics, IBM, Helsinki, Finland; 95% confidence interval [CI]). Intra‐assay repeatability was determined using 10 different reactions, and average of 31.5 Ct, 0.61 standard deviation with coefficient of variation of 2% for 894 copies and 35.6 Ct, 0.63 and 2% for 89 copies, respectively.

**TABLE 2:** Samples, controls and virus strains tested using WNV‐RT‐qPCR.

		No. of samples, reference result		WNV‐RT‐qPCR	
Panel/sample material	Microbial agent	WNV neg	WNV pos	Neg	Pos
**Serum**	WNVAb negatives^a^	14		14	0
	**Total**	**14**		**14**	**0**
**CSF**	WNVAb negatives^a^	5		5	0
	**Total**	**5**		**5**	**0**
**EQA** ^b^	Toscana virus (lineage A)	1		1	0
	Toscana virus (lineage B)	1		1	0
	TBEV	1		1	0
	WNV (lineage 1)		1	0	1
	WNV (lineage 2)		1	0	1
	Usutu virus	1		1	0
	Negatives (four plasma samples)	4		4	0
	**Total**	**8**	**2**	**8**	**2**
**WNV plasmid control** ^c^	WNV B956 (IDT); 8.9 × 10^5^–89 copies per reaction		50	0	50
	WNV B956 (IDT); 8.9 copies per reaction		10	5	5
	**Total**		**60**	**5**	**55**
**Virus nucleic acids** ^d^	WNV (strain Eg101; lin. 1, clade a); RNA stock dilutions 10^−^^1^–10^−^^4^		10	0	10
	WNV (strain B956; lineage 2); RNA stock dilutions 10^−^^1^–10^−^^4^		10	0	10
	WNV (in‐house; seq:strain Eg101; lin. 1, clade a); RNA stock dilutions 10^−^^1^–10^−^^4^		10	0	10
	WNV (strain 97–103); RNA stock dilutions 10^−^^1^–10^−^^4^		10	0	10
	YFV (strains 17D and Asibi)	2		2	0
	DENV1 (Zeptometrix)	1		1	0
	DENV2 (RKI)	1		1	0
	DENV3 (Zeptometrix)	1		1	0
	DENV4 (RKI)	1		1	0
	ZIKV (strain MR766)	1		1	0
	TBEV (European, Siberian and Far‐Eastern subtypes)	3		3	0
	CHIKV (strains Caribbean, EVAg, and Ross, in‐house)	2		2	0
	**Total**	**12**	**40**	**12**	**40**
**Altogether**	**WNV positives**		**102**	**5**	**97**
	**WNV negatives**	**39**		**39**	**0**
	**Concordance (%)**				**95.1%**
	**Specificity (%)**			**100**	

Abbreviations: CHIKV, chikungunya virus; DENV, dengue virus; neg, negative (no Ct‐value); No, number; pos, positive (Ct‐value <38); TBEV, tick‐borne encephalitis virus; USUV, Usutu virus; WNV, West Nile virus; YFV, yellow fever virus; ZIKV, Zika virus.

^a^ All CSF and serum samples tested for WNVAb in daily diagnostics between January 2021 and October 2023. All were negative for WNVAb (WNV IgG IFA, in‐house indirect immunofluorescence assay, and Anti‐WNV IgM ELISA, Euroimmun).

^b^ EVD‐LabNet (Ziegler et al. 2020) samples: Toscana virus (lineage A, 1.57 × 10^5^ RNA copies/0.4 mL), Toscana virus (lineage B, 1.24 × 10^5^ RNA copies/0.4 mL), Tick‐borne encephalitis virus (5.06 × 10^4^ RNA copies/0.4 mL), West Nile virus [lineage 1, strain UVE/WNV/2001/FR/DON2001 (ref#001V‐02215), 7.2 × 10^4^ RNA copies/0.4 mL], West Nile virus [lineage 2, strain B956, source BNI, Hamburg, 4.96 × 10^5^ RNA copies/0.4 mL], Usutu virus (6.34 × 10^3^ RNA copies/0.4 mL) and four negative plasma samples. All samples were extracted using the MagNA Pure LC system (Roche); 200 µL was extracted and eluted to 50 µL of elution buffer.

^c^ Plasmid: WNV B956 commercial plasmid (IDT; quantified).

^d^ WNV (Eg101 and B956; RNAs kindly provided by the Arbovirus and Imported Viral Diseases Laboratory, CNM, ISCIII, Instituto de Salud Carlos III, Centro Nacional de Microbiología). DENV1‐4 (ZeptoMetrix Corporation, Buffalo, New York, USA; RKI, Robert Koch Institute, kindly provided; Domingo et al. 2010). Viral control strains were extracted using QIAamp Viral RNA Mini Kit (Qiagen)).

The USUV‐RT‐qPCR has been validated by Cavrini et al. (2011), and in our study, we determined only the LOD using our PCR process. The LOD was 14.6 copies per reaction for USUV (isolate Budapest) using Probit Analysis (SPSS; CI 95%). Intra‐assay repeatability was determined using 10 different reactions, and average of 27.2 Ct, 0.23 standard deviation with coefficient of variation of 1% for 2493 copies and 30.1 Ct, 0.51 and 2% for 249 copies, respectively.

### 3.2 Molecular and Serological Screening of Bird and Human Patient Samples

All human and bird samples tested negative for WNV and USUV nucleic acids based on RT‐qPCR. Serological analysis using IFA confirmed that all bird samples were negative for WNV antibodies.

## 4 Discussion

Although WNV and USUV have not yet been detected in Finland, increasing concern arises as climate change drives their emergence and northward expansion in Europe, with a recent case of WNV reported in a bird in Latvia and Estonia (WOAH 2024; WOAH 2025). WNV has already been reported to overwinter in mosquitoes in Germany, and the Netherlands has also reported its first autochthonous cases of WNV (Ziegler et al. 2020; Vlaskamp et al. 2020). Experimental studies have demonstrated that native mosquito species in Finland and Sweden, *Culex pipiens* biotype *pipiens* and *Culex torrentium*, can efficiently transmit WNV and USUV under suitable temperature and humidity conditions (Krambrich et al. 2024; Jansen et al. 2023). These findings highlight the need for robust diagnostic tools to enable early detection and effective control measures.

Although WNV and USUV were not detected in either bird or human samples, we successfully established, optimized, and validated molecular diagnostic tools for the detection of these viruses. The sensitivity (LODs) and specificity (100%) were good (Table 2). No unspecific PCR results were observed during screening, further supporting assay reliability. Molecular diagnostics are essential for detecting WNV and USUV, particularly in regions where other flaviviruses such as TBEV are endemic. Serological assays for flaviviruses present a diagnostic challenge due to significant cross‐reactivity among anti‐flaviviral antibodies. In Finland, TBEV remains the only flavivirus known to circulate endemically, with new foci identified almost annually (Finnish Institute for Health and Welfare). If WNV becomes established in Finland, molecular assays would be critical for distinguishing WNV infections from TBEV. While neutralization assays remain the gold standard for differentiating flavivirus‐specific antibodies, they are labour‐intensive, time‐consuming and require specific laboratory facilities, making them unsuitable for routine diagnostics. Therefore, well‐validated molecular assays offer more tools for early detection of WNV infection besides serological assays.

To ensure preparedness, diagnostic methods for detecting WNV infection should be evaluated and validated under local conditions, particularly in regions where other endemic flaviviruses are present and differential diagnosis is essential. In Finland, in addition to TBEV, there is a history of mosquito‐borne viruses such as Sindbis virus (SINV) causing Pogosta disease, as well as Inkoo virus (INKV) and Chatanga virus. These pathogens should be considered in differential diagnostic workflows (Putkuri et al. 2016). Neurological manifestations associated with WNV infection can closely resemble those of TBEV, increasing the risk of misdiagnosis, especially when relying on serological assays that may produce false results. Moreover, WNV and SINV infections can both present with non‐specific symptoms such as fever, rash, and arthralgia. While SINV, an alphavirus, is the most common mosquito‐borne viral disease in Finland and can be distinguished from WNV through specific serological tests, cases suspected of SINV may test negative and not be further investigated for other arboviruses such as INKV or WNV.

The increasing risk of travel‐related WNV transmission through blood donation has raised significant concerns. In 2024, Finnish travellers made 7,200,000 trips abroad, including approximately 3,600,000 trips to WNV‐endemic areas in Central, Western, Eastern, and Southern Europe, as well as the Americas (Suomen virallinen tilasto (SVT) 2023). Due to the risk of asymptomatic WNV infection, concerns regarding WNV in blood donation have increased. In Finland, blood donation restrictions apply to individuals who have travelled to WNV‐endemic areas—such as Italy, mainland Greece, France, Northeast Germany and Berlin—between July and November for 28 days (Finnish Red Cross Blood Service 2025). However, despite reports of autochthonous WNV infections in the Netherlands and a WNV outbreak among birds in Poland in 2022 (Niczyporuk et al. 2023), current blood‐donation guidelines do not include travel‐related restrictions for these countries (Finnish Red Cross Blood Service 2025). In this study, we did not detect any acute WNV infections or antibodies against WNV amongst Finnish travellers.

There are several factors, including climatic and other factors, influencing the WNV and USUV transmission and the geographical spread. Temperature influences both vector development and the replication rates of the virus within the vectors (Tran et al. 2014; Lourenço et al. 2020; Shocket et al. 2020). WNV incidence is highest in regions where average summer temperatures range from 23°C to 26°C, and within these areas, heatwaves promote outbreaks (Jansen et al. 2023; Shocket et al. 2020; Petersen et al. 2022). For example, the largest WNV outbreak in Europe in 2018 was associated with the hottest summer on record at that time (Petersen et al. 2022). In Finland, temporary summertime transmission of WNV could occur in Finland in the future, as Finnish *Culex* mosquitoes can become infected with WNV at a mean temperature of 18°C, with transmission requiring at least 14 consecutive days of temperatures at or above 24°C (Jansen et al. 2023). This suggests that by the end of the century, WNV transmission could become possible, provided that other contributing factors align (Jansen et al. 2023). Bird migration has been identified as a key pathway for the spread of WNV in birds to new regions (Reed et al. 2003). It is plausible that WNV could be introduced into Finland via migratory birds, given the active flyways between Africa, Southern/Central Europe, and Finland (Olowu et al. 2023). The geographical range of both WNV and USUV is expanding northward, with recent records of WNV in birds in Latvia and Estonia, and USUV in Sweden (WOAH 2024; WOAH 2025; Olofsson et al. 2024; WOAH 2024). In the Netherlands, WNV antibodies were detected in Eurasian coots as early as 2014–2015, and by 2020, the first WNV‐positive birds and mosquitoes were reported, preceding the first report of human cases (Sikkema et al. 2020; Vlaskamp et al. 2020; Lim et al. 2018). In Germany, studies indicated that WNV is capable of overwintering in mosquitoes, indicating potential for local persistence (Ziegler et al. 2020; Pietsch et al. 2020; Kampen et al. 2021). USUV has been detected in both resident and migratory birds across Europe, most recently in Sweden in 2024, as well as in mosquitoes across Southern and Central Europe (Agliani et al. 2023; Olofsson et al. 2024; Garigliany et al. 2014; Höfle et al. 2013; Panagopoulou et al. 2024). In addition, USUV antibodies have been reported in equids from Germany, Poland, and Spain (Bażanów et al. 2018; Bażanów et al. 2021; Guerrero‐Carvajal et al. 2021). This study has some limitations. The sample sizes for both bird (*N* = 163) and human (*N* = 337) samples were relatively small. Bird samples were obtained from a wildlife hospital and primarily included deceased, diseased or euthanized individuals, which may not represent the broader wild bird population. Human serum samples were retrospectively collected from patients suspected of acute infection, introducing potential selection biases especially in relation to asymptomatic cases. Geographic coverage was limited to southern Finland, and temporal coverage was restricted to 2020 (birds) and summer–autumn 2021 (humans). We note that there is a need for expanded, prospective and geographically stratified surveillance in future studies.

Given the ongoing northward spread of WNV and USUV in Europe, Finland should be considered at risk for future emergence. Although neither WNV nor USUV has been detected in the country to date, the presence of competent mosquito vectors, potential future climatic suitability and major migratory bird flyways indicate plausible pathways for introduction. In this study, we successfully optimized and validated molecular diagnostic tools for the detection of WNV and USUV, thereby enhancing national preparedness for early identification and response.

Considering the potential for asymptomatic infections, especially in birds and humans, expanded and coordinated surveillance efforts will be essential, including bird, mosquito, and human blood donor monitoring. Priority measures include annual screening of migratory and resident birds, particularly high‐risk species along major flyways, by combining molecular assays with serological screening, allowing detection of both active and past infections. Seasonal monitoring of *Culex pipiens* biotype *pipiens* and *Culex torrentium*, especially in southern and coastal regions where summer temperatures support WNV replication, would facilitate early detection of vectors. Continued assessment of travel‐related WNV exposure among blood donors, including temporary screening during periods of increased regional activity, is also recommended. Adopting a national One Health strategy, including annual joint risk assessments, harmonized diagnostic protocols, predefined response actions, timely and robust disease risk modelling, as well as strengthened collaboration and information sharing with corridor countries in northern Europe, would enable early recognition of heightened transmission risk. Implementing such a framework would improve Finland's capacity to detect emerging WNV or USUV and strengthen long‐term preparedness under changing climatic and ecological conditions.

## Author Contributions

S.S. provided and coordinated collections of deceased bird specimens from the Korkeasaari Zoo Wildlife Rehabilitation Centre. R.J., R.N. and E.M.K. processed the original bird samples. A.J.J. developed the RT‐qPCR method for detecting WNV and USUV. M.T.S., R.N. and A.J.J. conducted RT‐qPCR screening of bird and human samples, and M.T.S. and VO conducted the IFA analysis of bird samples. R.J., E.M.K. and A.J.J. designed the study. R.J., M.T.S., E.M.K. and A.J.J. wrote the first version of the manuscript. All authors contributed to editing the manuscript. All authors read and approved the final version of the manuscript.

## Funding

This work was financially supported by HUS Diagnostic Center (Helsinki University Hospital, Helsinki, Finland; Grants TYH2021110, TYH2023102 and TYH2024104) and the University of Helsinki Faculty of Medicine Early Career Investigator Funding.

## Ethics Statement

The patient samples used in this study were anonymous and treated according to research permit (No 159/HUS/151/2022).

## Conflicts of Interest

The authors declare no conflicts of interest.

## Data Availability

The data that support the findings of this study are available on request from the corresponding author. The data are not publicly available due to privacy or ethical restrictions.
